# Lily WRKY factor LlWRKY22 promotes thermotolerance through autoactivation and activation of *LlDREB2B*

**DOI:** 10.1093/hr/uhac186

**Published:** 2022-08-25

**Authors:** Ze Wu, Ting Li, Xing Cao, Dehua Zhang, Nianjun Teng

**Affiliations:** Key Laboratory of Landscaping Agriculture, Ministry of Agriculture and Rural Affairs/Key Laboratory of Biology of Ornamental Plants in East China, National Forestry and Grassland Administration, College of Horticulture, Nanjing Agricultural University, Nanjing 210095, China; Jiangsu Graduate Workstation of Nanjing Agricultural University and Nanjing Oriole Island Modern Agricultural Development Co., Ltd, Nanjing 210043, China; College of Agriculture, Nanjing Agricultural University, Nanjing 210095, China; Key Laboratory of Landscaping Agriculture, Ministry of Agriculture and Rural Affairs/Key Laboratory of Biology of Ornamental Plants in East China, National Forestry and Grassland Administration, College of Horticulture, Nanjing Agricultural University, Nanjing 210095, China; Jiangsu Graduate Workstation of Nanjing Agricultural University and Nanjing Oriole Island Modern Agricultural Development Co., Ltd, Nanjing 210043, China; College of Architecture, Yantai University, Yantai, 264005, China; Key Laboratory of Landscaping Agriculture, Ministry of Agriculture and Rural Affairs/Key Laboratory of Biology of Ornamental Plants in East China, National Forestry and Grassland Administration, College of Horticulture, Nanjing Agricultural University, Nanjing 210095, China; Jiangsu Graduate Workstation of Nanjing Agricultural University and Nanjing Oriole Island Modern Agricultural Development Co., Ltd, Nanjing 210043, China; Key Laboratory of Landscaping Agriculture, Ministry of Agriculture and Rural Affairs/Key Laboratory of Biology of Ornamental Plants in East China, National Forestry and Grassland Administration, College of Horticulture, Nanjing Agricultural University, Nanjing 210095, China; Jiangsu Graduate Workstation of Nanjing Agricultural University and Nanjing Oriole Island Modern Agricultural Development Co., Ltd, Nanjing 210043, China

## Abstract

Most of WRKY transcription factors play important roles in plant development, protection against disease, and response to abiotic stress; however, their roles in lily are largely unknown. Transcriptome analysis in lily (*Lilium longiflorum*) led to the identification and isolation of a WRKY-IIe gene, *LlWRKY22*, which was found to be activated at high temperature and play a positive role in thermotolerance regulation. *LlWRKY22* expression was continuously activated by heat stress. We further found that LlWRKY22 protein localized to the nucleus and exhibited transactivation activity in both yeast and plant cells, and that its C terminus contributed to its transactivation activity. Meanwhile, overexpression of *LlWRKY22* in lily improved thermotolerance and activated the expression of heat-related *LlDREB2B* gene; however, silencing of *LlWRKY22* exerted the opposite effects. Further analysis revealed that LlWRKY22 directly activated the expression of *LlDREB2B* by binding to two tandem W-box elements on its promoter. Simultaneously, we also found that LlWRKY22 can directly bind its own promoter, thereby activating its own expression and forming a positive regulatory loop. Combined, our findings demonstrated that LlWRKY22 may be a new regulator of heat stress response and positively participates in the establishment of thermotolerance by activating itself and *LlDREB2B*.

## Introduction

Plants are sessile organisms, which renders them more sensitive to temperature changes, including those brought by global climate change [1, 2]. To cope with high-temperature environments and ensure their survival, plants have developed highly complex regulatory mechanisms, in which numerous transcription factors (TFs) play vital roles [3].

The WRKY protein family comprises a large number of plant-specific TFs characterized by the presence of one or two conserved N-terminal WRKY domains and a C-terminal zinc-finger-like motif (Cys_2_His_2_ or Cys_2_His/Cys) [4–6]. The WRKYs are classified into three distinct groups based on the number of WRKY domains and the characteristics of the zinc-finger motif [7, 8]. Group I members contain two WRKY domains, group II members contain one WRKY domain and the Cys2-His2 zinc-finger motif, and group III members contain one WRKY domain with different zinc-finger motifs (Cys_2_-His/Cys, Cys_2_-His_2_). The members in group II are further divided into five subgroups (a–e) [8]. In addition to the zinc-finger and WRKY motifs common to these TFs, some WRKY members also possess leucine-zipper domains, kinase domains, serine–threonine-rich regions, glutamine-rich regions, proline-rich regions, nuclear localization signals, or TIR-NBS-LRRs [9]. Such distinct structural organization allows WRKYs to exert distinct regulatory roles, whereby they either activate or inhibit the expression of target genes by binding W-box elements present on their promoters [10].

To date, the WRKY TFs have been found to function in plant defense responses, substance metabolism, hormone synthesis, signal transduction, trichome and embryo formation, seed dormancy, and senescence [11–21]. WRKY proteins also play roles in multiple abiotic stresses [8, 22]. Several studies investigating WRKYs have focused on their roles in response to drought, cold, and nutrient deficiency [23–29]. Nonetheless, whether and how many WRKYs participate in heat stress response (HSR) remains poorly understood. The group I WRKYs AtWRKY25, AtWRKY26, and AtWRKY33 in *Arabidopsis* redundantly regulate thermotolerance by activating heat shock proteins [30, 31]. The WRKY-IId member of *Arabidopsis*, *AtWRKY39*, which is induced by HS, positively regulates HSR by mediating the cooperation between the salicylic acid- and jasmonic acid-activated signaling pathways [32]. We have previously found that *LlWRKY39* is also induced by HS in lily, and functions positively in thermotolerance by activating the expression of *LlMBF1c* and interacting with LlCaM3 [33]. Although the roles of some WRKY-I or WRKY-IId members in thermotolerance have been investigated, those of others remain to be elucidated in detail.

In this study, a heat-inducible WRKY-IIe member, *LlWRKY22*, was isolated and identified from lily (*Lilium longiflorum*). The LlWRKY22 protein was found to be localized in the nucleus and exhibited transactivation activity in both yeast and plant cells. Moreover, overexpression of *LlWRKY22* increased the thermotolerance of lily, whereas silencing it exerted the opposite effect. Further analysis indicated that LlWRKY22 positively regulates thermotolerance via directly activating its own expression as well as that of *LlDREB2B*.

## Results

### Lily LlWRKY22 is a heat-inducible WRKY-IIe member

**Figure 1:**
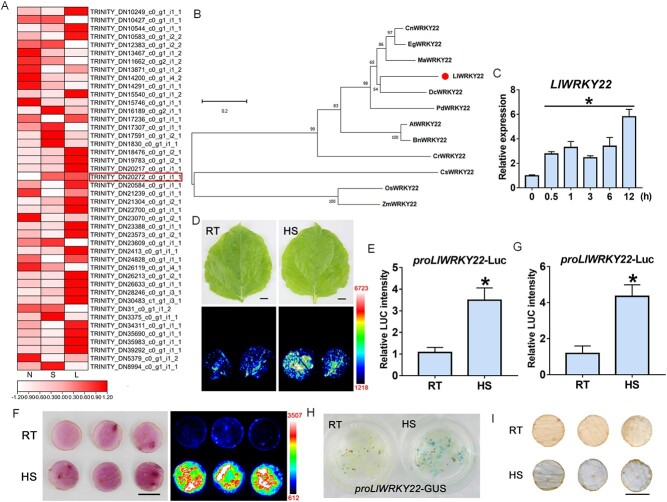
LlWRKY22 is a heat-inducible member of the WRKY-IIe subgroup. **A** Heat map of *WRKYs* expression in lily leaves with heat stress (HS). Heat map visualizing the expression pattern of *WRKYs* based on the heat-treated transcriptome of lily leaves, the red box indicates *LlWRKY22*. N: normal condition (22°C); S: exposed to 37°C for short time (1 h); L: exposed to 37°C for long time (10 h). **B** Phylogenetic tree analysis of LlWRKY22 and WRKY22 homologs from other species (bootstrap replicates, *n* = 1000). **C** The expression of *LlWRKY22* in lily leaves exposed to heat stress (HS; 37°C) of differing durations. Bars indicate means ± SD from three plants (Student’s *t*-test, ^*^P < 0.05, all treatments compared with 0 h). **D***LlWRKY22* promoter activity with the LUC reporter assay in tobacco leaves at room temperature (RT, 22°C) and under HS (37°C, 3 h; recovery at 22°C for 12 h). Representative image based on three independent experiments. Scale bar = 1 cm. **E** Quantitation of LUC intensity in tobacco leaves. All values are presented as means ± SD of three replicates (Student’s *t*-test, ^*^P < 0.05). **F***LlWRKY22* promoter activity with the LUC reporter assay in lily petal discs at room temperature (RT, 22°C) and under HS (37°C, 3 h; recovery at 22°C for 12 h). Representative image based on three independent experiments. Scale bar = 1 cm. **G** Quantitation of LUC intensity in lily petal discs. All values are presented as means ± SD of three replicates (Student’s *t*-test, ^*^P < 0.05). **H***LlWRKY22* promoter activity in *proLlWRKY22*-GUS transgenic *Arabidopsis* at RT (22°C) and under HS (37°C, 3 h). Representative image based on three independent experiments. **I***LlWRKY22* promoter activity in *proLlWRKY22*-GUS transiently expressed lily petal discs at RT (22°C) and under HS (37°C, 3 h). Representative image based on three independent experiments. Scale bar = 1 cm.

Analysis of the expression of *WRKY* genes in lily transcriptome revealed that *LlWRKY22* is a WRKY member differentially expressed under high-temperature conditions, *LlWRKY22* could be activated after a short time (1 h) or a long time (10 h) of HS (Fig. 1A). The open reading frame (ORF) of *LlWRKY22* was 840 bp and encoded a potential protein of 279 amino acids. Phylogenetic tree analysis using 72 WRKYs of *Arabidopsis* indicated that LlWRKY22 belongs to the WRKY-IIe subfamily and is closest to AtWRKY22 (Fig. S2, see online supplementary material). Accordingly, we named it LlWRKY22. Next, we explored the evolutionary relationship between LlWRKY22 and WRKY22 homologs from different species, including *Arabidopsis thaliana*, *Brassica napus*, *Cucumis sativus*, *Catharanthus roseus*, *Oryza sativa*, *Zea mays*, *Elaeis guineensis*, *Musa acuminate*, *Dendrobium catenatum*, *Cocos nucifera*, and *Phoenix dactylifera*. Based on the analysis of a phylogenetic tree created using the neighbor-joining method, the closest relationship was found between LlWRKY22 and DcWRKY22 of *D. catenatum*, both of which are non-grass monocots (Fig. 1B). Multiple sequence alignments of WRKY22 homologs from *Z. mays*, *O. sativa*, *P. dactylifera*, *E. guineensis*, *D. catenatum*, *M. acuminate*, and *A. thaliana* indicated that LlWRKY22 harbored a conserved WRKYGQ domain and a C2H2 (C-X5-C-X23-H-X1-H) zinc-finger motif at its C terminus (Fig. S2, see online supplementary material).

To detect *LlWRKY22* expression under high-temperature conditions, the tissue-cultured lily plants were exposed to 37°C for HS treatment. According to the RT-qPCR results, compared with treatment at 22°C, *LlWRKY22* expression was rapidly induced in the leaves after 0.5 h of HS, and stayed at a high level even 12 h after the HS treatment (Fig. 1C). Furthermore, the *LlWRKY22* promoter of 1053-bp was also cloned from lily genome, and the potential *cis*-elements were analysed with the online tool New PLACE (https://www.dna.affrc.go.jp/PLACE/?action=newplace). It was found that the *LlWRKY22* promoter contained many W-box elements (Table S1, see online supplementary material). This suggested *LlWRKY22* might be regulated by itself or other WRKY members. Subsequently, the promoter activity of *LlWRKY22* was analysed using a transient LUC reporter assay. We found that the signal from the *LlWRKY22* promoter-driven LUC reporter was significantly increased after HS treatment in tobacco leaves (Fig. 1D and E), and also in lily petal discs (Fig. 1F and G). Similarly, GUS activity in pro*LlWRKY22*-GUS transgenic seedlings was also increased after exposure to HS (Fig. 1H). In addition, the pro*LlWRKY22*-GUS was transiently transformed in the lily petal discs, and the GUS activity was also enhanced after HS, indicating that high temperature could activate the activity of *LlWRKY22* promoter in lily. These results suggested that LlWRKY22 was a heat-inducible member of WRKY-IIe subgroup.

### LlWRKY22 shows transactivation activity in yeast and plant cells

**Figure 2:**
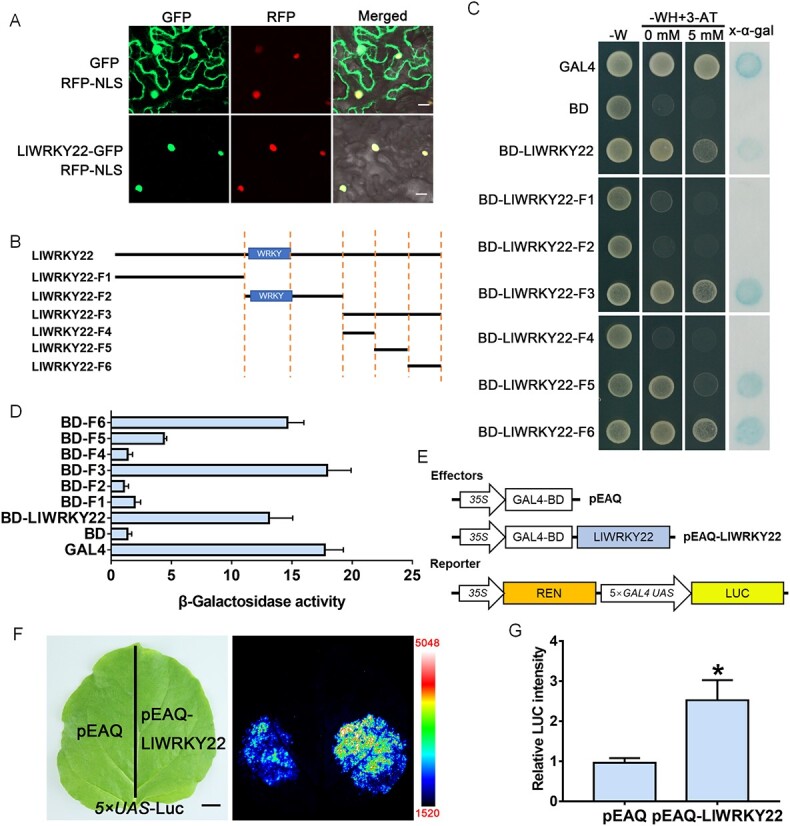
Subcellular localization and transactivation assay for LlWRKY22. **A** Detection of fluorescence signals in tobacco leaf cells co-transfected with LlWRKY22-GFP and the nuclear marker RFP-NLS. Scale bar = 50 μm. **B** Constructs used for the transactivation assay in yeast. **C** Transactivation activity assay in the yeast AH109 strain. The transformants were screened on SD-W medium (lacking Trp) while the growth of transformants was detected on SD-WH medium (lacking Trp/His) containing 3-amino-1,2,4-triazole (3-AT). The color reaction associated with x-α-gal degradation was used as a readout for β-galactosidase activity in the transformants. Representative image based on three replicates. **D** Determination of β-galactosidase activity using an enzymatic assay. Data are presented as means ± SD of three clones. **E** The constructs for the LUC reporter assay. Detection of the LUC signal in infiltrated tobacco leaves. The image is representative of three independent experiments. Scale bar = 1 cm. **F** Measurement of LUC intensity in the reporter assay (Student’s *t*-test, ^*^P < 0.05).

Transient expression of *LlWRKY22-GFP* in tobacco leaves showed that the GFP signal co-localized with that of the nuclear marker RFP-NLS, indicating that LlWRKY22 was a nuclear-acting protein (Fig. 2A). For the detection of transactivation activity, yeast AH109 cells were transformed with different vectors containing full-length or truncated LlWRKY22 fragments fused to the GAL4 DNA-binding domain (BD) (Fig. 2B). We found that transformants expressing the full-length LlWRKY22 protein propagated well on SD-WH medium (lacking Trp/His) and degraded x-α-gal (as detected by a color change), indicating that LlWRKY22 had transactivation ability in yeast cells (Fig. 2C). Analysis using truncated LlWRKY22 fragments revealed that the fragments F3, F5, and F6, but not F1, F2, and F4, possessed transactivation activity, demonstrating that the C terminus of LlWRKY22 contributed to its transactivation potential (Fig. 2C and D). In addition, using a LUC reporter system under the control of a 5 × *UAS* GAL4 promoter in tobacco leaves, we found that the LUC signal was stronger with BD-LlWRKY22 than with the BD-control (Fig. 2E–G). This result demonstrated that LlWRKY22 also exhibited transactivation ability in plant cells.

### *LlWRKY22* overexpression enhances the thermotolerance of lily

**Figure 3:**
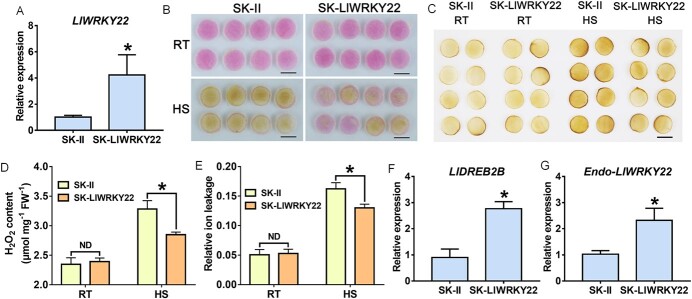
Thermotolerance assay using *LlWRKY22-*overexpressing petal discs. **A** Detection of *LlWRKY22* expression in *LlWRKY22*-overexpression petal discs. **B** The phenotypes of lily petal discs under normal conditions [room temperature (RT), 22°C] and after exposure to heat stress (HS, 40°C, 12 h). Representative image based on three experiments. Scale bar = 1 cm. **C** DAB staining of control and treated discs. Scale bar = 1 cm. **D** Determination of H_2_O_2_ content at 22°C (RT) and under HS (40°C, 12 h). Data are presented as means ± SD of three replicates (Student’s *t*-test, ^*^P < 0.05; ND, no significant difference; the SK-LlWRKY22 compared with the SK-II control under RT or HS condition, respectively). **E** Relative ion leakage (%) of discs at 22°C (RT) and under HS (40°C, 12 h). Data are presented as means ± SD of three replicates (Student’s *t*-test, ^*^P < 0.05; ND, no significant difference; the SK-LlWRKY22 compared with the SK-II control under RT or HS condition, respectively). **F**, **G** The expression of endogenous *LlDREB2B* and *LlWRKY22* in *LlWRKY22*-overexpression lily petal discs. Data are presented as means ± SD of three replicates (Student’s *t*-test, ^*^P < 0.05). DAB, 3,3′-diaminobenzidine; HS, heat stress; RT, room temperature.

To explore the role of LlWRKY22 *in vivo*, we overexpressed *LlWRKY22* in lily petals via transient transformation (Fig. 3A). After HS treatment, it was found that the petals transiently overexpressing *LlWRKY22* showed less fading compared with the controls (Fig. 3B). In addition, overexpression of *LlWRKY22* in petal discs did not affect the relative ion leakage and H_2_O_2_ content at 22°C (Fig. 3C–E); however, after HS, the values for both parameters were significantly higher in control discs than in *LlWRKY22*-overexpression discs (Fig. 3D and E). These results suggested that *LlWRKY22* overexpression protected lily cells from HS and increased their thermotolerance. According to previous studies, *DREB2-like* genes have been reported to be regulated by WRKY-I TFs in response to drought stress [34, 35]. Meanwhile, our previous study showed that the *DREB2-like* gene *LlDREB2B* of lily plays an important role in the establishment of thermotolerance [36]. Based on these data, it was speculated that *LlDREB2B* might be regulated by LlWRKY22. Therefore, we then detected the expression of *LlDREB2B* in *LlWRKY22*-overexpression petal discs, and the results showed that *LlWRKY22* overexpression could activate the expression of *LlDREB2B* (Fig. 3F). Furthermore, overexpression of *LlWRKY22* also led to a significant increase in the expression of endogenous *LlWRKY22* in lily (Fig. 3G).

### *LlWRKY22* silencing reduces the thermotolerance of lily

**Figure 4:**
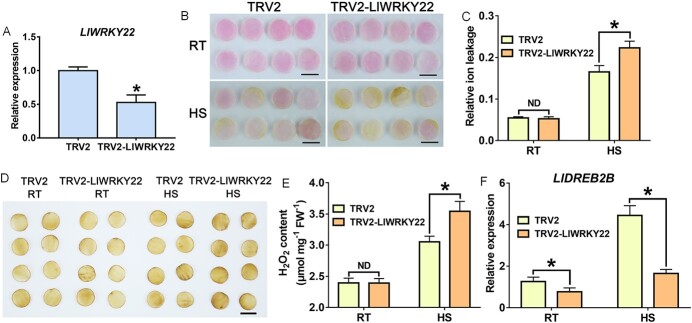
Thermotolerance assay in *LlWRKY22-*silenced petal discs. **A** The expression of *LlWRKY22* in TRV-VIGS lily petals. **B** The phenotypes of lily petal discs under normal conditions (RT, 22°C) and after HS (40°C, 12 h). Representative image based on three experiments. Scale bar = 1 cm. **C** Relative ion leakage (%) of discs at RT and under HS (40°C, 12 h). Data are presented as means ± SD of three replicates (Student’s *t*-test, ^*^P < 0.05; ND, no significant difference; TRV2-LlWRKY22 compared with the TRV2-control under RT or HS condition, respectively). **D** DAB staining of control and treated discs. Scale bar = 1 cm. **E** Determination of H_2_O_2_ content. Data are presented as means ± SD of three replicates (Student’s *t*-test, ^*^P < 0.05; ND, no significant difference; TRV2-LlWRKY22 compared with the TRV2-control under RT or HS condition, respectively). **F** The expression of *LlDREB2B* in petal discs subjected to TRV-VIGS at RT (22°C) and under HS (40°C, 3 h). Data are presented as means ± SD of three replicates (Student’s *t*-test, ^*^P < 0.05, the TRV2-LlWRKY22 compared with the TRV2-control under RT or HS condition, respectively). DAB, 3,3′-diaminobenzidine; HS, heat stress; RT, room temperature.

To further investigate the role of LlWRKY22 in thermotolerance of lily, the expression of *LlWRKY22* was silenced by TRV (tobacco rattle virus) -VIGS in petal discs (Fig. S3, see online supplementary material). With RT-qPCR analysis, the *LlWRKY22* was specially silenced with a lower level in gene expression compared with TRV2-control (Fig. 4A), and the three other WRKY members (*LlWRKY25*, *LlWRKY33*, and *LlWRKY39*) showed similar expression levels in control and silenced groups (Fig. S4, see online supplementary material). As determined using a VIGS assay, petals deficient for *LlWRKY22* showed more fading after HS treatment compared with that seen in the TRV2 controls (Fig. 4B). Moreover, silencing of *LlWRKY22* in petal discs did not affect the relative ion leakage at 22°C; however, after HS, the relative ion leakage values of TRV2-control discs were lower than those of TRV2-LlWRKY22 discs (Fig. 4C), and the similar result was also seen for the H_2_O_2_ content (Fig. 4D and E). These data suggested that silencing of *LlWRKY22* worsens the HS-induced damage in lily cells and decreases thermotolerance. Further RT-qPCR analysis showed that silencing of *LlWRKY22* led to a significant reduction in the expression of *LlDREB2B* in lily compared with the unsilenced controls (Fig. 4F). These findings indicated that the accumulation of LlWRKY22 might be required for the heat-inducible expression of *LlDREB2B*.

### LlWRKY22 binds to the promoter of *LlDREB2B* and activated its expression

**Figure 5:**
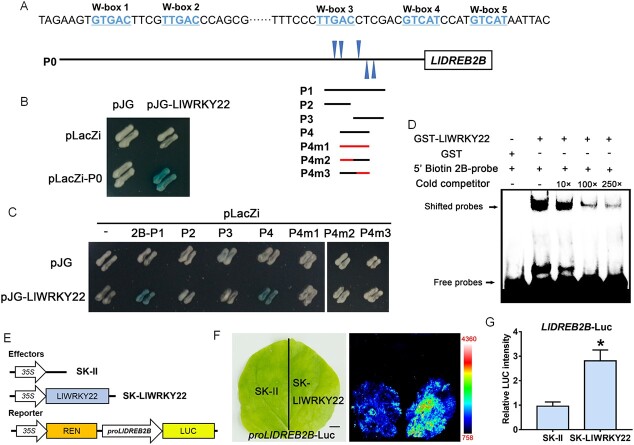
LlWRKY22 activates the expression of *LlDREB2B*. **A** Diagram of the *LlDREB2B* promoter. The W-box elements are marked with blue triangles. The truncated fragments used for the yeast one-hybrid (Y1H) assay are marked with black lines. The mutant fragment used for the Y1H assay is marked with a red line. **B** A Y1H assay for LlWRKY22 and the promoter of *LlDREB2B*. Representative image based on three replicates. **C** A Y1H assay for LlWRKY22 and the *LlDREB2B* promoter fragments. Fragment activity was analysed by a color change on Ura−/Trp-deficient SD medium following the addition of x-gal. Representative image based on three replicates. **D** An electrophoretic mobility shift assay (EMSA) of GST-LlWRKY22 and the W-box elements from the *LlDREB2B* promoter. Representative image based on three experiments. **E** Constructs used in the dual-luciferase reporter assay. **F** Detection of the LUC signal in tobacco leaves. Representative image based on three experiments. Scale bar = 1 cm. **G** Measurement of LUC intensity in the dual-luciferase reporter assay. Data are presented as means ± SD of three replicates (Student’s *t*-test, ^*^P < 0.05).

Since *LlDREB2B* expression was activated in *LlWRKY22*-overexpression lily petals (Fig. 3F), we speculated that LlWRKY22 might directly regulate its expression. The results of yeast one-hybrid (Y1H) assay showed that LlWRKY22 bound to the *LlDREB2B* promoter (2B-P0) (Fig. 5A and B). Meanwhile, we identified five conserved W-box elements in the P1 promoter region of *LlDREB2B* (2B-P1) (Fig. 5A), and further Y1H assay found that LlWRKY22 bound to this region (Fig. 5C). Next, we truncated the 2B-P1 region into three fragments (2B-P2, 2B-P3, and 2B-P4) and performed a Y1H assay using these fragments (Fig. 5A). The results demonstrated that LlWRKY22 bound to fragment 2B-P4, but not 2B-P2 or 2B-P3 (Fig. 5C), and analysis of the 2B-P4 fragment revealed the presence of two tandem W-box elements (TTGAC). Then, we found that LlWRKY22 could not bind to the mutant 2B-P4 fragments in which both W-box elements (2B-P4m1) or either one (2B-P4m2 and 2B-P4m3) were mutated (TAAAC) (Fig. 5A and C). The EMSA result also demonstrated that GST-LlWRKY22 could bind to the tandem W-box elements in the *LlDREB2B* promoter, indicating that LlWRKY22 might exert direct regulatory effects on*LlDREB2B* (Fig. 5D; Table S2, see online supplementary material). In addition, the results of the dual-luciferase reporter assay further indicated that LlWRKY22 could enhance the activity of the *LlDREB2B* promoter (Fig. 5E–G), which suggested that LlWRKY22 might directly activate *LlDREB2B* via binding to its promoter.

### LlWRKY22 activates its own expression

**Figure 6:**
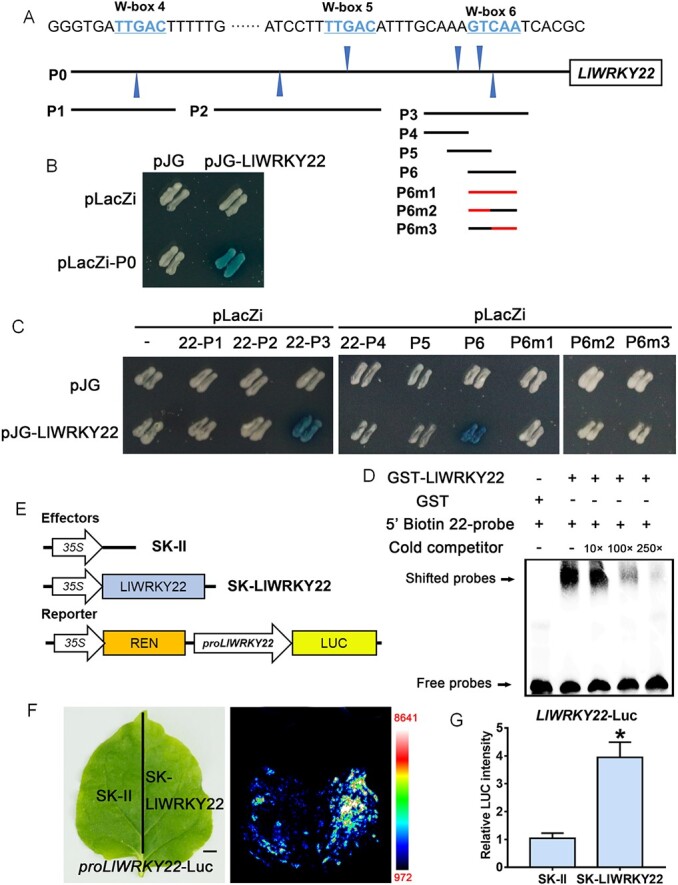
LlWRKY22 directly activates its own expression. **A** Diagram of the *LlWRKY22* promoter. The W-box elements are marked with blue triangles. The truncated fragments used for the yeast one-hybrid (Y1H) assay are marked with black lines. The mutant fragment used for the Y1H assay is marked with a red line. **B** A Y1H assay for LlWRKY22 and its own promoter. Representative image based on three replicates. **C** A Y1H for LlWRKY22 and the fragments of the *LlWRKY22* promoter. Fragment activity was analysed by a color change on Ura−/Trp-deficient SD medium following the addition of x-gal. Representative image based on three replicates. **D** An electrophoretic mobility shift assay (EMSA) for GST-LlWRKY22 and the tandem W-box elements from its own promoter. Representative image based on three experiments. **E** Constructs used in the dual-luciferase reporter assay. **F** Detection of the LUC signal in tobacco leaves. Representative image based on three experiments. Scale bar = 1 cm. **G** Measurement of LUC intensity in the dual-luciferase reporter assay. Data are presented as means ± SD of three replicates (Student’s *t*-test, ^*^P < 0.05).

Many WRKY members have been reported to display positive or negative self-regulation [8]. Here, we found that the *LlWRKY22* promoter contains six conserved W-box elements, suggesting that LlWRKY22 might also have self-regulatory activity (Fig. 6A). Using a Y1H assay, we found that LlWRKY22 could bind to its own promoter (22-P0) (Fig. 6B). Truncation analysis indicated that LlWRKY22 recognized a fragment harboring three W-box (TTGAC) elements (22-P3), but not another two fragments containing one W-box element each (22-P1 and 22-P2) (Fig. 6C). A Y1H assay using fragments of the 22-P3 region (22-P4, 22-P5, and 22-P6) further showed that LlWRKY22 could bind fragment 22-P6, but not fragments 22-P4 and 22-P5 (Fig. 6C). The 22-P6 fragment contained two tandem W-box elements; however, LlWRKY22 could not bind to a mutant 22-P6 fragment (22-P6m1) in which the two tandem W-box elements were mutated (TAAAC), and also not bind to the mutant 22-P6 fragments (22-P6m2 and 22-P6m3) in which either one W-box element was mutated (Fig. 6C). An EMSA assay also confirmed that GST-LlWRKY22 recognized and bound the two tandem W-box elements from the *LlWRKY22* promoter, which indicated that LlWRKY22 might directly regulate its own expression (Fig. 6D; Table S2, see online supplementary material). Dual-luciferase assay results showed that LlWRKY22 could activate its own promoter activity (Fig. 6E–G). Simultaneously, overexpression of *LlWRKY22* in lily petals also led to a significant increase in the expression of endogenous *LlWRKY22* (Fig. 3F). Combined, these findings suggested that LlWRKY22 may be involved in a positive self-regulatory loop.

## Discussion

In *Arabidopsis*, AtWRKY22 is implicated in pathogen-triggered immunity, anti-aphid responses, dark-induced senescence, and response to oxidase stress [37–40]. However, no study to date has reported a role for WRKY22 in thermotolerance. Transcriptome data analysis indicated that *LlWRKY22* was a heat-inducible gene expressed in lily leaves. Here, we isolated and identified LlWRKY22, which contains a classical WRKY domain and belongs to the WRKY-IIe subgroup. We further uncovered that *LlWRKY22* expression was activated by high temperature, and that LlWRKY22 protein was localized in the nucleus, where it displayed transactivation activity and exerted its positive effects in response to HS.

Studies in *Arabidopsis* have indicated that three WRKY-I subfamily members, AtWRKY25, AtWRKY26, and AtWRKY33, play positive roles in thermotolerance [31]. The genes encoding these proteins exhibit distinct expression patterns under HS conditions, with *AtWRKY25* and *AtWRKY26* showing increased expression and *AtWRKY33* decreased expression. Additionally, while their mutants display reduced thermotolerance, the opposite is observed in transgenic plants overexpressing any of these genes [30, 31]. These three WRKY proteins participate in the HSR by modulating the transcriptional reprogramming of heat-responsive genes and positively regulating the cooperation between the ethylene- and heat shock protein-related signaling pathways [22, 31]. AtWRKY39, a member of the WRKY-IId subgroup, has also been reported to be involved in thermotolerance, and its expression can be rapidly induced by high temperature [32]. Studies have reported that *WRKY22* can be induced in response to oxidative stress or hypoxia in *Arabidopsis* [37, 39] as well as by pathogenic bacteria in *Arabidopsis*, rice, peach, and citrus [41–46]. In rice, *OsWRKY22* is inducible by and positively regulates tolerance to aluminum [47]. Our results showed that the expression of *LlWRKY22* was activated by HS (Fig. 1), which suggested it may be involved in the regulation of thermotolerance.

At the protein level, LlWRKY22 was found to be distributed in the nucleus, which was consistent with the localization reported in its homologs [44, 46], suggesting that it may act as a TF (Fig. 2). WRKY proteins have been described as having the capacity to act as activators, repressors, or both, as well as to contain both transcriptional activation and repression domains [6]. For instance, AtWRKY6, AtWRKY33, and AtWRKY53 can either activate or inhibit the expression of target genes in a promoter sequence-dependent manner [48–50]. Our results revealed that the C terminus of LlWRKY22 was a transactivation domain, and that LlWRKY22 exhibits transactivation activity in both yeast and tobacco cells, suggesting that it was able to function as a *trans*-activator (Fig. 2).

DREB2-like TFs, such as AtDREB2A, AtDREB2B, and AtDREB2C, are widely reported to be involved in stress responses, especially in the regulation of drought and heat tolerance [3, 51]. AtHSFA1s, a key HSR-related factor in *Arabidopsis*, can directly regulate the expression of *AtDREB2A* [52]. Furthermore, both *AtDREB2A* and *AtDREB2C* can act upstream of *AtHSFA3* to directly activate its expression and participate in the HSR [53–56]. *AtDREB2A* can also function as a downstream gene of AtMBF1c, an important stress signal-bridging factor [57]. These observations demonstrate that DREB2s are important regulators of HSR and actively participate in the establishment of thermotolerance. We have previously shown that the lily DREB2 homolog, LlDREB2B, plays an important positive role in thermotolerance, and that the DREB2-HSFA3 module is also conserved in lily [36]. In response to drought stress, *DREB2-like* genes have been reported to be involved in the regulatory pathway of WRKY-I members. For example, overexpression of wheat WRKY-I members *TaWRKY2* and *TaWRKY19* in *Arabidopsis* activates the expression of *AtDREB2A* to improve its drought and salt tolerances [34]; the WRKY-I member *GhWRKY59* of cotton is induced by drought stress and directly activates *GhDREB2* expression to promote drought tolerance [35]. However, it has not been reported whether other WRKY members regulate *DREB2-like* genes in response to HS. In this study, we found that the WRKY-IIe member LlWRKY22 directly promotes the expression of *LlDREB2B* via binding to its promoter (Fig. 5). *LlWRKY22* overexpression in lily stimulated the expression of *LlDREB2B* (Fig. 3), while silencing of *LlWRKY22* significantly inhibited the heat-inducible expression of *LlDREB2B* (Fig. 4), which suggested the potential existence of a WRKY22-DREB2-HSFA3 regulatory pathway. It has been reported that some WRKY proteins preferentially recognize two tandem W-box elements [46]. Our analysis indicated that the *LlDREB2B* promoter contains five W-box elements, and we demonstrated that LlWRKY22 could bind to the two tandem W-boxes in fragment 2B-P4 (Fig. 5). In rice, OsWRKY22 directly binds to two tandem W-box elements in the promoter of the citrate transporter gene *OsFRDL4*, thereby activating its expression and positively regulating rice tolerance to aluminum [47]. Meanwhile, MsWRKY22 of alfalfa was shown to bind to two tandem W-box elements in the promoter of *MsWRKY11*, thereby activating its expression and enhancing drought tolerance [29]. Furthermore, citrus CsWRKY22 was shown to activate the expression of *CsLOB1*, a key gene involved in the regulation of susceptibility to citrus canker, by binding two tandem W-box elements in the *CsLOB1* promoter, thereby negatively affecting resistance to the *Xanthomonas citri* pathogen [46].

Here, we also found that LlWRKY22 directly activates its own expression by binding to two tandem W-box elements on its promoter, thus forming a self-activating loop (Fig. 6). Many studies have shown that WRKY proteins display extensive auto-regulatory and cross-regulatory activity [6]. For example, AtWRKY53, which has a role in leaf senescence, inhibits its own expression by binding to its own promoter, thereby forming a negative feedback regulatory loop [49]. In contrast, AtWRKY33, which induces camalexin biosynthesis and has a role in defense responses, can be self-activated by binding to its own promoter, and thus generate a positive feedback regulatory loop [58]. Likewise, pepper CaWRKY40 participates in responses to pathogen attack and HS through activating its own expression by binding to two tandem W-box elements in its promoter [59]. Our results showed that LlWRKY22 positively regulates itself, amplifies the high-temperature signal, and enhances thermotolerance.

Overall, our results indicated that LlWRKY22 is a WRKY-IIe member that is activated under HS conditions and is involved in thermotolerance by activating its own expression and that of the key regulator *LlDREB2B*.

## Materials and methods

### Plant materials and growth conditions

Sterile tissue-cultured *L. longiflorum* plantlets of the thermotolerant cv. ‘White heaven’ were used in this study. The plantlets were cultured on MS medium at 22°C under a 16-h/8-h light/dark photoperiod. After sterilization and washing, *Arabidopsis thaliana* (Col) and *Nicotiana benthamiana* (tobacco) seeds were sowed and germinated on MS medium. After 10 days of germination, the seedlings were individually transplanted into plastic pots and grown in an incubator (22/16°C, 16-h/8-h light/dark photoperiod).

### Cloning of *LlWRKY22* from lily ‘white heaven’

Total RNA was extracted from the leaves of 2-week-old, tissue-cultured lily plantlets subjected to HS for 1 h at 37°C. The cDNA was synthesized from total RNA using a kit of HiScript II Q Select RT SuperMix (+gDNA wiper) (R233–01, Vazyme, China). Based on our transcriptome data, the ORF of *LlWRKY22* was amplified using the designed primers shown in Table S3, see online supplementary material.

### Phylogenetic tree analysis and multiple sequence alignment

Phylogenetic trees were generated in MEGA 7.0 using the neighbor-joining method. Multiple sequence alignment of WRKY22 homologs from different plant species was performed with BioEdit 7.0 in conjunction with ClustalW 2.0 software.

### Isolation of the *LlWRKY22* and *LlDREB2B* promoters

The sequence of the *LlDREB2B* promoter has been previously reported [36]. The method of HiTail-PCR [60] was used to isolate the promoter of *LlWRKY22*. The 1053-bp fragment upstream of the start codon (ATG) of *LlWRKY22* was cloned and identified from the genome of lily ‘White heaven’.

### Transient overexpression assay in lily petals

The method of transient transformation of lily petals has been reported in previous studies [61–63], and we have performed the experiment according to these procedures with little modifications in this study. Cultures of bacteria expressing SK-II or SK-LlWRKY22 were resuspended in an infiltration buffer (10 mM MES, 200 μM acetosyringone, 10 mM MgCl_2_, pH 5.6) and then placed at 22°C for 5 h without light. Unopened flowers of similar size (~10 cm in length) of the lily cv. ‘Sorbonne’ were used for transient overexpression. First, the outer petals were removed, and then discs (1 cm in diameter) were excised from the inner petals with a hole punch. The discs were subsequently immersed in bacterial solutions and infiltrated under negative pressure (−0.7 MPa, 15 min). The infiltrated discs were washed with sterile water and placed on an agar plate (0.4%) for 96 h at 22°C. For HS treatment, the discs were exposed to 40°C for 12 h. The color fading of petal discs was observed and recorded. The HS treatment would cause the accumulation of intracellular peroxides, which oxidize pigments and prompt their degradation, the less thermotolerant materials showed more quick color fading after exposed to HS; therefore, the reduction of pigments can reflect the thermotolerance of plants [64–67]. The H_2_O_2_ content and relative ion leakage were determined both for treated and untreated petal discs. Simultaneously, the H_2_O_2_ content in the control and treated discs were also detected using DAB staining. The sequences of the primers used for vector construction are shown in Table S4, see online supplementary material.

### Silencing of *LlWRKY22* in lily petals using virus-induced gene silencing (VIGS)

The TRV-VIGS was performed according to the procedures described previously [62] with little modifications. A 303-bp fragment of *LlWRKY22* was amplified by PCR and inserted into the TRV2 vector to generate TRV2-LlWRKY22. Then, TRV1, TRV2, and TRV2-LlWRKY22 were respectively introduced into *Agrobacterium tumefaciens* GV3101. The bacterial cultures were resuspended in the above-mentioned infiltration buffer. A mixture of bacterial solutions containing equal ratios (*v*/*v*, OD_600_ = 1.0) of TRV1 and TRV2 or TRV1 and TRV2-LlWRKY22 were used for the infiltration of TRV2-control and TRV2-LlWRKY22 petal discs, respectively (Table S5, see online supplementary material). The mixtures of bacterial solutions were infiltrated into the lily petal discs as described above. After 5 days, the discs were subjected to HS, and then harvested for determination of ion leakage and H_2_O_2_ content. The H_2_O_2_ contents of the control and treated discs were also detected using DAB staining.

### Analysis of *LlWRKY22* promoter activity

To analyse promoter activity under HS, the 1053-bp promoter of *LlWRKY22* was inserted into the pGreenII0800-LUC and pCAMBIA1391-GUS vectors, yielding *proLlWRKY22*-LUC and *proLlWRKY22*-GUS, respectively. The reconstructed vectors were respectively transformed into the *A. tumefaciens* GV3101 (pSoup) strain. A solution of transformed bacteria harboring *proLlWRKY22*-LUC was injected into tobacco leaves; after 48 h, the tobacco leaves were subjected to HS (37°C for 3 h followed by 12 h of recovery at 22°C), and subsequently cut to observe the LUC signal. The *proLlWRKY22*-LUC was also transiently transformed into lily petal discs as described above, and after 48 h, the discs was exposed to HS (37°C for 3 h followed by 12 h of recovery at 22°C). The LUC intensity was measured as previously described using Andor Solis v15 software [33]. The *A. tumefaciens* cells harboring *proLlWRKY22*-GUS were stably transformed into *Arabidopsis* and transiently transformed into lily petal discs. The 7-day-old transgenic seedlings and the petal discs were subjected to HS treatment (37°C for 3 h) and then sampled for the GUS assay.

### Transactivation analysis of LlWRKY22

The full-length and truncated fragments of *LlWRKY22* were cloned into the pGBKT7 yeast expression vector [designed to express a fusion protein of the GAL4 DNA-binding domain (DNA-BD)]. GAL4 served as a positive control and empty BD as a negative control. For the analysis of transactivation activity, the yeast AH109 strain was transformed with these vectors, and the transformants were grown on SD minimal yeast medium. The *LlWRKY22* ORF was cloned into the pEAQ vector to produce BD-LlWRKY22 proteins. The empty pEAQ vector was used as a negative control. A *5 × GAL4 UAS* element and a mini 35S promoter were fused and inserted into pGreenII0800-LUC. These reconstructed vectors were separately transformed into *A. tumefaciens* GV3101 (pSoup). A mixture of bacterial suspensions was infiltrated into tobacco leaves for the transactivation analysis. The LUC signal of the tobacco leaves was observed and imaged using a CCD camera after 48 h of infiltration and LUC activity was quantified.

### Subcellular localization analysis of LlWRKY22-GFP

The *LlWRKY22* ORF without a stop codon was cloned into the pCAMBIA1300-GFP vector to generate a LlWRKY22-GFP fusion protein. RFP-NLS was used as a nuclear marker. Reconstructed and empty vectors were individually transformed into *A. tumefaciens* GV3101. For co-localization analysis, a mixture of bacteria respectively harboring LlWRKY22-GFP and RFP-NLS was injected into tobacco leaves. After 48 h of infiltration, the leaves were cut, and GFP and RFP fluorescence were observed and imaged using a laser scanning confocal microscope (LSM800, Zeiss, Germany).

### Heat treatments and *LlWRKY22* expression assay

The healthy, 2-week-old, tissue-cultured ‘White heaven’ plantlets were exposed to HS of differing duration (0, 0.5, 1, 3, 6, and 12 h) at 37°C in a thermostatic incubator (Liance, China). The leaves of HS-treated plants were sampled for *LlWRKY22* expression using reverse transcription-quantitative PCR (RT-qPCR). The lily 18S rRNA was used for normalization. Details of the primers used for qPCR are shown in Table S6, see online supplementary material.

### Y1H assay

The *LlDREB2B* and *LlWRKY22* promoter fragments were separately cloned into the pLacZi vector. The *LlWRKY22* ORF was inserted into the pJG vector, yielding pJG-LlWRKY22, with the empty pJG vector serving as a negative control. Yeast EGY48 strain cells were co-transformed with these reconstructed vectors for the Y1H assay. Transformants were screened by growing them on Ura−/Trp-deficient SD medium in an incubator at 30°C. Binding activity was analysed on Ura−/Trp-deficient SD medium via a color change following the addition of x-gal.

### Electrophoretic mobility shift assay (EMSA)

The *LlWRKY22* ORF was cloned into the pGEX-4 T-1 vector to generate GST-LlWRKY22 fusion proteins. The GST-LlWRKY22 proteins were synthesized in *Escherichia coli* BL21 cells via the addition of isopropyl β-D-1-thiogalactopyranoside (IPTG). The accumulation of GST-LlWRKY22 proteins was detected by sodium dodecyl sulfate–polyacrylamide gel electrophoresis (SDS–PAGE). The GST-LlWRKY22 and GST proteins were enriched using a Pierce GST Spin Purification Kit (Thermo Fisher, New York, NY, USA). The EMSA probes were labeled with biotin at the 5′-end and the EMSA was performed with an EMSA kit (Thermo Fisher).

### Dual-luciferase reporter assay

The *LlWRKY22* ORF was cloned into the pGreenII 62-SK (SK-II) vector to generate the SK-LlWRKY22 effector vector, with the empty vector serving as a negative control. The *LlDREB2B* and *LlWRKY22* promoters were cloned into pGreenII0800-LUC to generate LUC reporter vectors. These vectors were individually transformed into *A. tumefaciens* GV3101 (pSoup) cells and mixtures of bacteria expressing different combinations of reporters and effectors were injected into tobacco leaves. The LUC signal of the infiltrated leaves was observed after 48 h of infiltration and the LUC intensity was measured.

## Acknowledgements

This work was supported by National Natural Science Foundation of China (31902055), and the Natural Science Foundation of Jiangsu Province, China (BK20190532), the National Key R&D Program of China (2019YFD1000400).

## Author contributions

N.T. and Z.W. conceived and designed the experiments; Z.W., T.L., and D.Z. performed the experiments under the supervision of N.T.; X.C. provided technical help; Z.W. and T.L. analysed the data; Z.W. wrote the manuscript; all the authors revised and approved the final version of the manuscript.

## Data availability

The data underlying this article will be shared on reasonable request to the corresponding author.

## Conflict of interests

The authors declare no conflict of interests.

## Supplementary data

Supplementary data is available at *Horticulture Research * online.

## Supplementary Material

Web_Material_uhac186
