# Supplementary material for: Lily WRKY factor LlWRKY22 promotes thermotolerance through autoactivation and activation of LlDREB2B
Source: Hortic Res. 2022 Aug 25;9:uhac186. doi: 10.1093/hr/uhac186 (PMC9627522; doi:10.1093/hr/uhac186)
Supplement: Web_Material_uhac186 [file web_material_uhac186.zip › WRKY22 supplemental figures.docx]

**
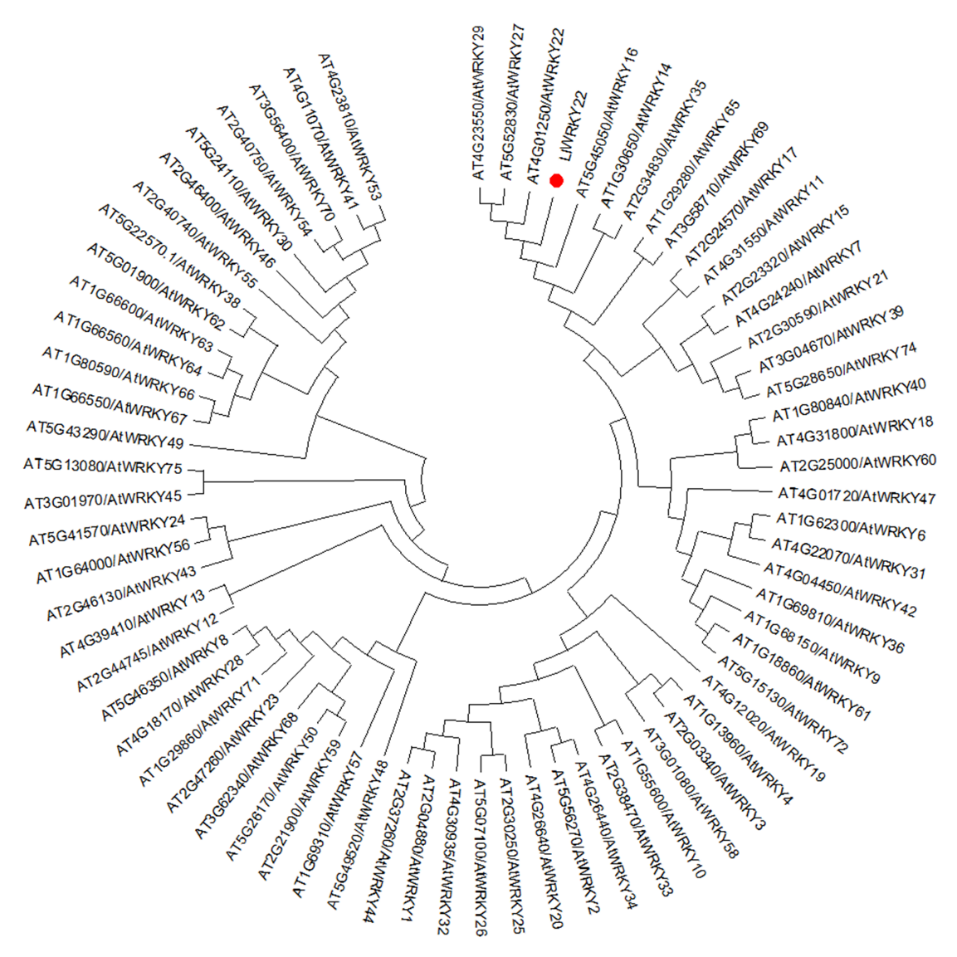
**

**Fig. S1.** Phylogenetic analysis of LlWRKY22 and all WRKY members of Arabidopsis. The evolutionary tree was assembled in MEGA 7.0. Node values are percentages of bootstraps generated, with *n* = 1,000 bootstrap replicates.


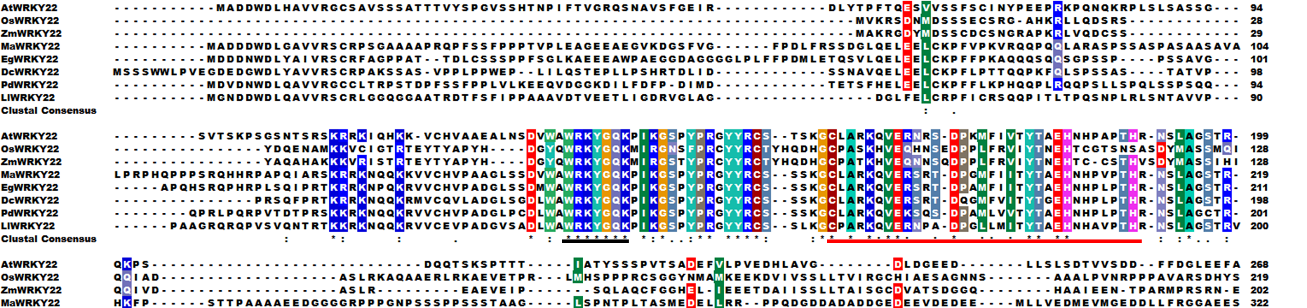


**Fig. S2.** Protein alignment of LlWRKY22 and its homologs from different plant species. AtWRKY22, *Arabidopsis thaliana*, AT4G01250; EgWRKY22, *Elaeis guineensis*, XP_010915790.1; PdWRKY22, *Phoenix dactylifera*, XP_008792407.2; MaWRKY22, *Musa acuminata*, XP_009409443.1; DcWRKY22, *Dendrobium catenatum*, XP_020684026.1; OsWRKY22, *Oryza sativa*, DAA05087.1; ZmWRKY22, *Zea mays*, NP_001147816.1. The conserved WRKY motif is labeled with a black line. The red line indicates the C2H2 motif of LlWRKY22.


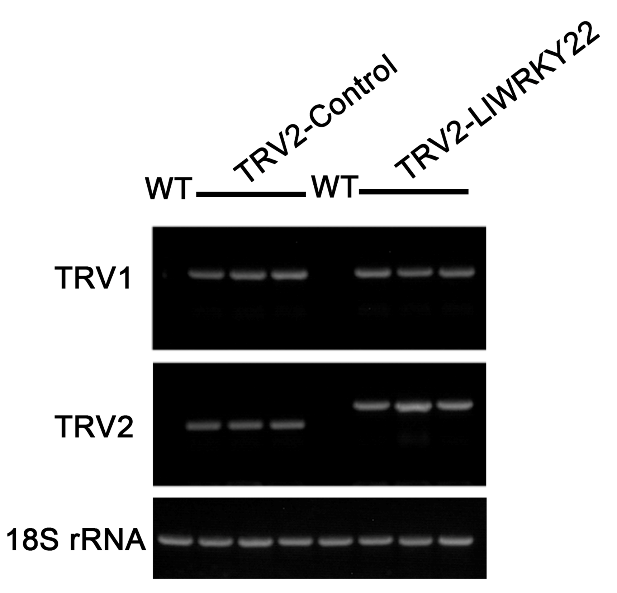


**Fig. S3.** Molecular analysis of VIGS lily petals by RT-PCR. The infiltrated petal discs of TRV2-control and TRV2-LlWRKY22 were used to detect TRV1 and TRV2, respectively. PCR of TRV1 and TRV2 was performed with 35 cycles. Lily 18S rRNA was used for normalization, 30 cycles were performed.


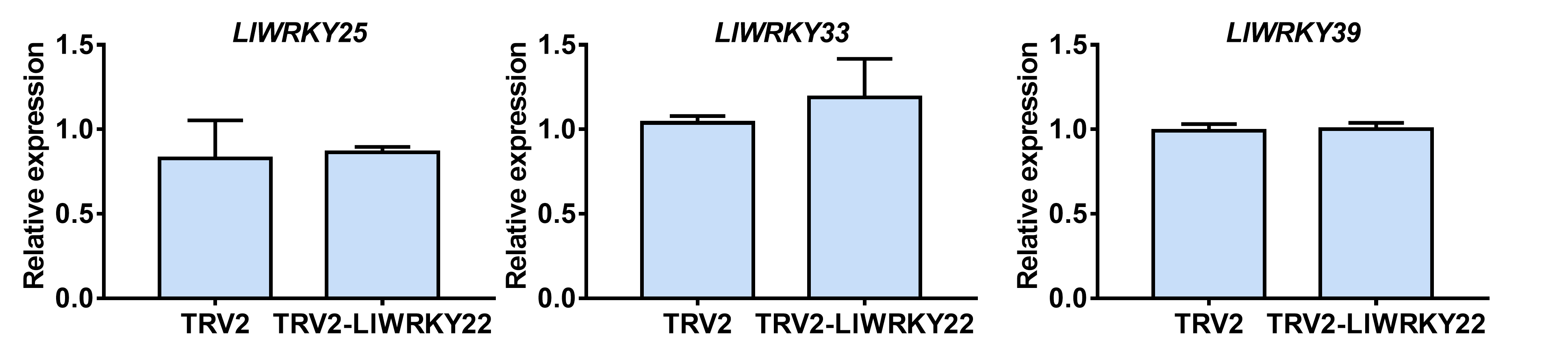


**Fig. S4.** The expression analysis of *LlWRKY25*, *LlWRKY33*, and *LlWRKY39* in TRV-VIGS lily petal discs. Lily 18S rRNA was used for normalization. Data are presented as means ± SD of three replicates.
