# Supplementary material for: Lily WRKY factor LlWRKY22 promotes thermotolerance through autoactivation and activation of LlDREB2B
Source: Hortic Res. 2022 Aug 25;9:uhac186. doi: 10.1093/hr/uhac186 (PMC9627522; doi:10.1093/hr/uhac186)
Supplement: Web_Material_uhac186 [file web_material_uhac186.zip › WRKY22 Supplemental Tables.docx]

**Supplemental Table S1.** Distribution of the potential *cis*-elements in the *LlWRKY22* promoter.

| **Factor or Site Name** | **Loc.(Str.)** | **Factor or Site Name** | **Loc.(Str.)** |
| --- | --- | --- | --- |
| ARR1AT | 5(-)NGATT | MARTBOX | 506(-)TTWTWTTWTT |
| GATABOX | 22(+)GATA | MARTBOX | 507(-)TTWTWTTWTT |
| ROOTMOTIFTAPOX1 | 23(+)ATATT | MARTBOX | 508(-)TTWTWTTWTT |
| CAATBOX1 | 32(+)CAAT | MARTBOX | 509(-)TTWTWTTWTT |
| POLASIG2 | 35(-)AATTAAA | CAATBOX1 | 518(-)CAAT |
| MYBPZM | 42(+)CCWACC | RAV1AAT | 520(-)CAACA |
| SEF3MOTIFGM | 44(+)AACCCA | WBOXATNPR1 | 522(+)TTGAC |
| CANBNNAPA | 47(+)CNAACAC | WBOXHVISO1 | 523(+)TGACT |
| RAV1AAT | 48(+)CAACA | WRKY71OS | 523(+)TGAC |
| CACTFTPPCA1 | 51(+)YACT | WBOXNTERF3 | 523(+)TGACY |
| GTGANTG10 | 60(+)GTGA | NODCON2GM | 550(-)CTCTT |
| RAV1AAT | 66(+)CAACA | OSE2ROOTNODULE | 550(-)CTCTT |
| AACACOREOSGLUB1 | 67(+)AACAAAC | ARR1AT | 559(+)NGATT |
| ANAERO1CONSENSUS | 70(+)AAACAAA | POLLEN1LELAT52 | 571(-)AGAAA |
| AACACOREOSGLUB1 | 71(+)AACAAAC | DOFCOREZM | 574(-)AAAG |
| ANAERO1CONSENSUS | 74(+)AAACAAA | GT1CONSENSUS | 575(-)GRWAAW |
| AACACOREOSGLUB1 | 75(+)AACAAAC | GT1GMSCAM4 | 575(-)GAAAAA |
| TBOXATGAPB | 89(+)ACTTTG | POLLEN1LELAT52 | 577(-)AGAAA |
| DOFCOREZM | 90(-)AAAG | DOFCOREZM | 580(-)AAAG |
| NODCON2GM | 95(-)CTCTT | POLLEN1LELAT52 | 582(-)AGAAA |
| OSE2ROOTNODULE | 95(-)CTCTT | GT1CONSENSUS | 592(-)GRWAAW |
| CACTFTPPCA1 | 98(-)YACT | RAV1AAT | 604(+)CAACA |
| GTGANTG10 | 99(+)GTGA | CPBCSPOR | 614(-)TATTAG |
| ROOTMOTIFTAPOX1 | 106(-)ATATT | POLASIG3 | 616(+)AATAAT |
| -300ELEMENT | 110(+)TGHAAARK | ROOTMOTIFTAPOX1 | 619(-)ATATT |
| DOFCOREZM | 113(+)AAAG | ROOTMOTIFTAPOX1 | 624(+)ATATT |
| CIACADIANLELHC | 126(+)CAANNNNATC | CACTFTPPCA1 | 631(-)YACT |
| DOFCOREZM | 127(+)AAAG | GT1MOTIFPSRBCS | 634(+)KWGTGRWAAWRW |
| POLLEN1LELAT52 | 129(+)AGAAA | GT1CONSENSUS | 638(+)GRWAAW |
| ARR1AT | 132(-)NGATT | TATABOX5 | 640(-)TTATTT |
| ARR1AT | 139(-)NGATT | POLASIG2 | 649(+)AATTAAA |
| REALPHALGLHCB21 | 149(+)AACCAA | CPBCSPOR | 656(-)TATTAG |
| DOFCOREZM | 154(+)AAAG | POLASIG3 | 658(+)AATAAT |
| NODCON2GM | 155(-)CTCTT | ARR1AT | 661(-)NGATT |
| OSE2ROOTNODULE | 155(-)CTCTT | EECCRCAH1 | 671(-)GANTTNC |
| WBBOXPCWRKY1 | 158(-)TTTGACY | ARR1AT | 674(-)NGATT |
| WBOXHVISO1 | 158(-)TGACT | CACTFTPPCA1 | 682(+)YACT |
| WBOXNTERF3 | 158(-)TGACY | IBOXCORENT | 693(-)GATAAGR |
| WBOXATNPR1 | 159(-)TTGAC | IBOX | 694(-)GATAAG |
| WRKY71OS | 159(-)TGAC | IBOXCORE | 695(-)GATAA |
| CIACADIANLELHC | 161(+)CAANNNNATC | GATABOX | 696(-)GATA |
| MYB1AT | 162(+)WAACCA | NODCON1GM | 697(-)AAAGAT |
| REALPHALGLHCB21 | 163(+)AACCAA | OSE1ROOTNODULE | 697(-)AAAGAT |
| CCAATBOX1 | 165(+)CCAAT | DOFCOREZM | 699(-)AAAG |
| CAATBOX1 | 166(+)CAAT | POLLEN1LELAT52 | 701(-)AGAAA |
| ARR1AT | 167(-)NGATT | RHERPATEXPA7 | 705(-)KCACGW |
| CACTFTPPCA1 | 172(-)YACT | CACTFTPPCA1 | 733(+)YACT |
| CAATBOX1 | 175(-)CAAT | ARFAT | 745(-)TGTCTC |
| SEF4MOTIFGM7S | 187(-)RTTTTTR | SEBFCONSSTPR10A | 745(-)YTGTCWC |
| CIACADIANLELHC | 187(+)CAANNNNATC | SURECOREATSULTR11 | 745(+)GAGAC |
| CAATBOX1 | 201(+)CAAT | GTGANTG10 | 758(+)GTGA |
| PYRIMIDINEBOXOSRAMY1A | 212(-)CCTTTT | ARR1AT | 759(+)NGATT |
| DOFCOREZM | 213(+)AAAG | CAATBOX1 | 761(-)CAAT |
| GATABOX | 220(+)GATA | WBOXATNPR1 | 762(+)TTGAC |
| PYRIMIDINEBOXHVEPB1 | 225(-)TTTTTTCC | WBOXHVISO1 | 763(+)TGACT |
| GT1CONSENSUS | 225(+)GRWAAW | WRKY71OS | 763(+)TGAC |
| GT1CONSENSUS | 226(+)GRWAAW | WBOXNTERF3 | 763(+)TGACY |
| GT1GMSCAM4 | 226(+)GAAAAA | DOFCOREZM | 766(-)AAAG |
| POLLEN1LELAT52 | 240(+)AGAAA | CACTFTPPCA1 | 773(-)YACT |
| GT1CONSENSUS | 241(+)GRWAAW | POLLEN1LELAT52 | 777(-)AGAAA |
| GT1CONSENSUS | 245(-)GRWAAW | GATABOX | 797(-)GATA |
| IBOXCORE | 246(-)GATAA | MYBST1 | 797(-)GGATA |
| SREATMSD | 246(+)TTATCC | PYRIMIDINEBOXOSRAMY1A | 800(+)CCTTTT |
| GATABOX | 247(-)GATA | DOFCOREZM | 801(-)AAAG |
| MYBST1 | 247(-)GGATA | WBOXATNPR1 | 804(+)TTGAC |
| DOFCOREZM | 255(+)AAAG | BIHD1OS | 805(-)TGTCA |
| ARR1AT | 269(+)NGATT | WRKY71OS | 805(+)TGAC |
| EECCRCAH1 | 270(+)GANTTNC | EBOXBNNAPA | 808(-)CANNTG |
| PROLAMINBOXOSGLUB1 | 274(+)TGCAAAG | MYCCONSENSUSAT | 808(-)CANNTG |
| DOFCOREZM | 277(+)AAAG | EBOXBNNAPA | 808(+)CANNTG |
| POLLEN1LELAT52 | 282(+)AGAAA | MYCCONSENSUSAT | 808(+)CANNTG |
| GT1CONSENSUS | 283(+)GRWAAW | -300ELEMENT | 812(+)TGHAAARK |
| INRNTPSADB | 285(-)YTCANTYY | PROLAMINBOXOSGLUB1 | 812(+)TGCAAAG |
| CAATBOX1 | 287(-)CAAT | TBOXATGAPB | 814(-)ACTTTG |
| CPBCSPOR | 294(-)TATTAG | DOFCOREZM | 815(+)AAAG |
| POLASIG1 | 296(+)AATAAA | WBOXHVISO1 | 817(-)TGACT |
| AACACOREOSGLUB1 | 305(+)AACAAAC | WBOXNTERF3 | 817(-)TGACY |
| ANAERO1CONSENSUS | 308(+)AAACAAA | WBOXATNPR1 | 818(-)TTGAC |
| DOFCOREZM | 313(+)AAAG | WRKY71OS | 818(-)TGAC |
| CACTFTPPCA1 | 338(-)YACT | CAATBOX1 | 820(+)CAAT |
| ARR1AT | 345(-)NGATT | ARR1AT | 821(-)NGATT |
| NODCON2GM | 362(-)CTCTT | GTGANTG10 | 823(-)GTGA |
| OSE2ROOTNODULE | 362(-)CTCTT | ABRERATCAL | 824(+)MACGYGB |
| SURECOREATSULTR11 | 364(+)GAGAC | CGCGBOXAT | 825(-)VCGCGB |
| POLLEN1LELAT52 | 374(+)AGAAA | CGCGBOXAT | 825(+)VCGCGB |
| GT1CONSENSUS | 375(+)GRWAAW | E2FANTRNR | 827(-)TTTCCCGC |
| GT1GMSCAM4 | 375(+)GAAAAA | E2FCONSENSUS | 827(-)WTTSSCSS |
| CAATBOX1 | 381(+)CAAT | E2F1OSPCNA | 827(+)GCGGGAAA |
| ARR1AT | 382(-)NGATT | GT1CONSENSUS | 836(+)GRWAAW |
| RAV1AAT | 385(+)CAACA | GT1GMSCAM4 | 836(+)GAAAAA |
| DOFCOREZM | 392(+)AAAG | ARR1AT | 840(-)NGATT |
| NODCON2GM | 393(-)CTCTT | ARR1AT | 845(+)NGATT |
| OSE2ROOTNODULE | 393(-)CTCTT | GTGANTG10 | 855(+)GTGA |
| WBBOXPCWRKY1 | 396(-)TTTGACY | ACGTATERD1 | 860(-)ACGT |
| WBOXHVISO1 | 396(-)TGACT | ACGTATERD1 | 860(+)ACGT |
| WBOXNTERF3 | 396(-)TGACY | TBOXATGAPB | 866(+)ACTTTG |
| WBOXATNPR1 | 397(-)TTGAC | DOFCOREZM | 867(-)AAAG |
| WRKY71OS | 397(-)TGAC | CCAATBOX1 | 884(+)CCAAT |
| INRNTPSADB | 405(-)YTCANTYY | LEAFYATAG | 884(+)CCAATGT |
| CAATBOX1 | 407(-)CAAT | CAATBOX1 | 885(+)CAAT |
| CACTFTPPCA1 | 411(-)YACT | CACTFTPPCA1 | 892(+)YACT |
| TATABOX5 | 418(-)TTATTT | MYBPLANT | 904(+)MACCWAMC |
| POLASIG1 | 419(+)AATAAA | ANAERO1CONSENSUS | 908(+)AAACAAA |
| DPBFCOREDCDC3 | 426(+)ACACNNG | AACACOREOSGLUB1 | 909(+)AACAAAC |
| CACTFTPPCA1 | 427(+)YACT | SV40COREENHAN | 911(-)GTGGWWHG |
| CARGCW8GAT | 439(-)CWWWWWWWWG | MYB1AT | 912(+)WAACCA |
| CARGCW8GAT | 439(+)CWWWWWWWWG | ARR1AT | 930(+)NGATT |
| DOFCOREZM | 445(+)AAAG | CAREOSREP1 | 941(+)CAACTC |
| ARFAT | 470(-)TGTCTC | CACTFTPPCA1 | 949(+)YACT |
| SEBFCONSSTPR10A | 470(-)YTGTCWC | DOFCOREZM | 951(-)AAAG |
| SURECOREATSULTR11 | 470(+)GAGAC | DOFCOREZM | 958(-)AAAG |
| TATABOX5 | 475(-)TTATTT | DOFCOREZM | 976(+)AAAG |
| POLASIG3 | 476(+)AATAAT | WBOXHVISO1 | 978(-)TGACT |
| ARR1AT | 479(-)NGATT | WBOXNTERF3 | 978(-)TGACY |
| CAATBOX1 | 482(+)CAAT | WBOXNTCHN48 | 978(-)CTGACY |
| SORLIP2AT | 488(-)GGGCC | WRKY71OS | 979(-)TGAC |
| CCAATBOX1 | 492(+)CCAAT | CTRMCAMV35S | 988(+)TCTCTCTCT |
| CAATBOX1 | 493(+)CAAT | CTRMCAMV35S | 990(+)TCTCTCTCT |
| ARR1AT | 494(-)NGATT | CTRMCAMV35S | 992(+)TCTCTCTCT |
| GTGANTG10 | 496(-)GTGA | GT1CONSENSUS | 1003(-)GRWAAW |
| MARTBOX | 501(-)TTWTWTTWTT | POLLEN1LELAT52 | 1005(-)AGAAA |
| MARTBOX | 502(-)TTWTWTTWTT | GATABOX | 1019(+)GATA |
| MARTBOX | 503(-)TTWTWTTWTT | GATABOX | 1021(-)GATA |
| MARTBOX | 504(-)TTWTWTTWTT | MYBST1 | 1021(-)GGATA |
| MARTBOX | 505(-)TTWTWTTWTT | REBETALGLHCB21 | 1021(-)CGGATA |

**Supplemental Table S2.** The promoter sequences are used for EMSA assay.

| **Promoter fragments** | **Sequences** |
| --- | --- |
| 2B-probe | ACTTCGTTGACCCAGCGGAGACAGATAGGTGAGACTGCCGAAGGCCGCTCGTGGTCGAGTTTCCCTTGACCTCGAC |
| 22-probe | CTAGAGCGGTGCTCCTCTATCCTTTTGACATTTGCAAAGTCAATCACGCGGGAAACGAAAAATCCGGA |

**Supplemental Table S3.** Primers of *LlWRKY22* isolation.

| **Primer name** | **Sequences** |
| --- | --- |
| *LlWRKY22-*ORF-F | 5’-ATGGGGAACGATGATTGGGATCT-3’ |
| *LlWRKY22-*ORF-R | 5’-TCAAAGAGTGATGTCGTCAAAGAAT-3’ |

**Supplemental Table S4.** Primers used for vector reconstruction.

| **Plasmid name** | **Vectors** | **Primer sequences** |
| --- | --- | --- |
| pCAMBIA1300-GFP | GFP-LlWRKY22 | 5’-CTCGGCATGGACGAGCTGTACAAGGTCGAC ATGGGGAACGATGATTGGGATCT-3’  5’-ATGTTTGAACGATCGGGGAAATTCGAGCTC TCAAAGAGTGATGTCGTCAAAGAAT-3’ |
| pCAMBIA1391 | *proLlWRKY22*-GUS | 5’-TTGGGCCCGGCGCGCCAAGCTTGGCTGCAG  GATTAATCATAAACTTGAGCTGA-3’  5’-GTGGACTCCTCTTAGAATTCCCGGGGATCC  GAGGAGAGAAGACAAACAATAGCTC-3’ |
| pGBKT7 | BD-LlWRKY22 | 5’-GACCTGCATATGGCCATGGAGGCCGAATTC ATGGGGAACGATGATTGGGATCT-3’  5’-GGGTTATGCTAGTTATGCGGCCGCTGCAG TCAAAGAGTGATGTCGTCAAAGAAT-3’ |
| pGBKT7 | BD-F1 | 5’-GACCTGCATATGGCCATGGAGGCCGAATTC ATGGGGAACGATGATTGGGATCT-3’  5’-GGGTTATGCTAGTTATGCGGCCGCTGCAG  TCACGAGACTCCATCTGCGGGCAC-3’ |
| pGBKT7 | BD-F2 | 5’-GACCTGCATATGGCCATGGAGGCCGAATTC  GCCGATTTGTGGGCTTGGCGCA-3’  5’-GGGTTATGCTAGTTATGCGGCCGCTGCAG  TCAGCTGCCGGCGAGGGAGTTGCGGT-3’ |
| pGBKT7 | BD-F3 | 5’-GACCTGCATATGGCCATGGAGGCCGAATTC  ACCCGAGTTCAGAAGCTGCCGTCA-3’  5’-GGGTTATGCTAGTTATGCGGCCGCTGCAG TCAAAGAGTGATGTCGTCAAAGAAT-3’ |
| pGBKT7 | BD-F4 | 5’-GACCTGCATATGGCCATGGAGGCCGAATTC  ACCCGAGTTCAGAAGCTGCCGTCA-3’  5’-GGGTTATGCTAGTTATGCGGCCGCTGCAG TCACTCCTCACTCTTGCACGGCCTGTTG-3’ |
| pGBKT7 | BD-F5 | 5’-GACCTGCATATGGCCATGGAGGCCGAATTC GAGGCAGAGTCGGAGGAGCTCCTGGT-3’  5’-GGGTTATGCTAGTTATGCGGCCGCTGCAG TCAACCGAGGAATAACAAATCGTCCTCG-3’ |
| pGBKT7 | BD-F6 | 5’-GACCTGCATATGGCCATGGAGGCCGAATTC GGGGAGGTCGAGGGCCAGCCCAGT-3’  5’-GGGTTATGCTAGTTATGCGGCCGCTGCAG TCAAAGAGTGATGTCGTCAAAGAAT-3’ |
| pEAQ-BD | pBD- LlWRKY22 | 5’-AGACAGTTGACTGTATCGCCGACCGGT ATGGGGAACGATGATTGGGATCT-3’  5’-ATTTAATGAAACCAGAGTTAAAGGCCT TCAAAGAGTGATGTCGTCAAAGAAT-3’ |
| pJG | pJG- LlWRKY22 | 5’-GATGTGCCAGATTATGCCTCTCCCGAATTC ATGGGGAACGATGATTGGGATCT-3’  5’-CTCTGGCGAAGAAGTCCAAAGCTTCTCGAG TCAAAGAGTGATGTCGTCAAAGAAT-3’ |
| pLacZi | pLacZi-LlWRKY22 | 5’-ATCATTTCCTTTGATATTGGATCGGAATTC  GATTAATCATAAACTTGAGCTGA-3’  5’-TTTATATACATACAGAGCACATGCCTCGAG  GAGGAGAGAAGACAAACAATAGCTC-3’ |
| pLacZi | pLacZi-LlDREB2B | 5’-ATCATTTCCTTTGATATTGGATCGGAATTCTGG  AGGGTTCGATCGATTATGAG-3’  5’-TTTATATACATACAGAGCACATGCCTCGAGCGT  GCAAAGATCTCAGTTTTGGACTG-3’ |
| pGreenII 0800-LUC | *ProLlWRKY22*-LUC | 5’-GGCGAATTGGGTACCGGGCCCCCCCTC  GAGGATTAATCATAAACTTGAGCTGA-3’  5’-GGTGGCGGCCGCTCTAGAACTAGTGGATCC  GAGGAGAGAAGACAAACAATAGCTC-3’ |
| pGreenII 0800-LUC | *ProLlDREB2B*-LUC | 5’-GGCGAATTGGGTACCGGGCCCCCCCTCGAGTGG  AGGGTTCGATCGATTATGAG-3’  5’-GGTGGCGGCCGCTCTAGAACTAGTGGATCCCGT  GCAAAGATCTCAGTTTTGGACTG-3’ |
| pTRV2 | pTRV2-LlWRKY22 | 5’-TCTGTGAGTAAGGTTACCGAATTCTCTA  GACAAGGTGGTGCGGCAACTAGGGATA-3’  5’- CCGGGCCTCGAGACGCGTGAGCTCGGT  ACCTTCACACACCACCCTCTTCTGCTGGT-3’ |

**Supplemental Table S5.** RT-PCR primers for detection of VIGS-LlWRKY22 lily plants.

| **Gene name** | **Sequences** |
| --- | --- |
| *TRV1* | 5’-TTACAGGTTATTTGGGCTAG-3’  5’-CCGGGTTCAATTCCTTATC-3’ |
| *TRV2* | 5’-TGGGAGATGATACGCTGTT-3’  5’-CCTAAAACTTCAGACACG-3’ |

**Supplemental Table S6.** RT-qPCR primers.

| **Gene name** | **Sequences** |
| --- | --- |
| *LlWRKY22* | 5’-GCGAGGACGATTTGTTATTCCTC-3’  5’-GTGATGTCGTCAAAGAATGCAG-3’ |
| *Endogenous LlWRKY22* | 5’-CAGTGGCTCAACATCCAGTG-3’  5’-TTGGATCTGATTCTCACTTCCAC-3’ |
| *LlDREB2B* | 5’-CTTTGCAGGGAGGGAGCTTGTTCT-3’  5’-ACTAGCAGCATACTAGCCTAATCCCT-3’ |
| *LlWRKY25* | 5’-GAGAAACTATGGATTGTCCGGGTACGA-3’  5’-AAACAATTCATCCTCCCTCGGTTCCT-3’ |
| *LlWRKY33* | 5’-CGGATGTTGCAGCAGAACCAAGGGATG-3’  5’-AGGCCAGCCATGAGTCTAGAAGCAAGT-3’ |
| *LlWRKY39* | 5’-CTTGGAGGAAATATGGGCAGAAGCC-3’  5’-GTGTTAGTAGCTTGGGGTGG-3’ |
| *18S rRNA* | 5’-AGTTGGTGGAGCGATTTGTCT-3’  5’-CCTGTTATTGCCTCAAACTTCC-3’ |
